# Chemical Fingerprint of ‘Oblačinska’ Sour Cherry (*Prunus* *cerasus* L.) Pollen

**DOI:** 10.3390/biom9090391

**Published:** 2019-08-21

**Authors:** Milica Fotirić Akšić, Uroš Gašić, Dabić Zagorac Dragana, Milica Sredojević, Tomislav Tosti, Maja Natić, Mekjell Meland

**Affiliations:** 1 Faculty of Agriculture, University of Belgrade, Nemanjina 6, 11080 Belgrade, Serbia; 2 Faculty of Chemistry, University of Belgrade, Studentski trg 16, 11000 Belgrade, Serbia; 3 University of Belgrade, Innovation Center, Faculty of Chemistry, Studentski trg 16, 11000 Belgrade, Serbia; 4 Norwegian Institute of Bioeconomy Research—NIBIO Ullensvang, 5781 Lofthus, Norway

**Keywords:** clone, carbohydrates, ‘Oblačinska’ sour cherry, phenolics, pollen

## Abstract

The aim of this research was to analyze sugars and phenolics of pollen obtained from 15 different ‘Oblačinska’ sour cherry clones and to assess the chemical fingerprint of this cultivar. Carbohydrate analysis was done using high-performance anion-exchange chromatography (HPAEC) with pulsed amperometric detection (PAD), while polyphenols were analyzed by ultra-high-performance liquid chromatography–diode array detector–tandem mass spectrometry (UHPLC-DAD MS/MS) system. Glucose was the most abundant sugar, followed by fructose and sucrose. Some samples had high level of stress sugars, especially trehalose. Rutin was predominantly polyphenol in a quantity up to 181.12 mg/kg (clone III/9), with chlorogenic acid (up to 59.93 mg/kg in clone III/9) and *p*-coumaric acid (up to 53.99 mg/kg in clone VIII/1) coming after. According to the principal component analysis (PCA), fructose, maltose, maltotriose, sorbitol, and trehalose were the most important sugars in separating pollen samples. PCA showed splitting off clones VIII/1, IV/8, III/9, and V/P according to the quantity of phenolics and dissimilar profiles. Large differences in chemical composition of studied ‘Oblačinska sour cherry’ clone pollen were shown, proving that it is not a cultivar, but population. Finally, due to the highest level of phenolics, clones IV/8, XV/3, and VIII/1 could be singled out as a promising one for producing functional food and/or in medicinal treatments.

## 1. Introduction

Pollen, a microgametophyte in seeded plants, is one of the most important reproductive plant products because it carries spermatic cells, which are necessary in double fertilization and, thus, in population sustainability. It develops in androeceum, where tapetum cells regulate sugar transport in the whole anther mostly nourishing pollen grains [1]. During flowering time pollen grains are fully developed having reserves that are necessary for pollen germination and fusion with a female gamete [2,3]. When it falls on a sticky stigma, a pollen tube starts to grow through the transmitting tissue of the pistil (using sugars and energy), and if the crossing combination is compatible it reaches the embryo sac that contains the egg cell.

The chemistry of pollen grains differs due to the botanical and geographic origin, edaphic and environmental parameters (temperature, water, and light intensity), and type of pollination and pollinizers [4]. Carbohydrates, mostly polysaccharides (starch, callose, pectin, cellulose, and sporopollenin) and low molecular sugars (fructose, glucose, and sucrose) are one of the important components of pollen grains and constitute between 13 and 55% of pollen [5,6]. Content of starch can be in range from 3.6 to 13%, hydrated short-lived pollen grains have high level, and dry long-lived pollen grains have very low level (but high in sucrose) [7]. Pollen grains store high level of minerals, which can vary from 2.5 to 6.5% of pollen dry weight. The highest portion goes to nitrogen (0.36% to 9.7%), while other important minerals are K, Mg, P, Ca, S, B, Zn, Cu, Mn, and Fe [8]. Pollen grains are a rich source of amino acids, and the six most common are aspartic acid, glutamic acid, proline, leucine, lysine, and arginine, comprising 60% of protein. Proline is the most abundant and can account for 1 to 2% of the whole pollen grain weight [9]. Protein is estimated from 10 to 40% (and directly influences the bee body’s largeness), while being poorest in anemoflimous gymnosperms [10].

Pollen has up to 10% of lipids, where fatty acids are nutritionally important for pollinators [11]. Anemomphilous plants have the lowest level of lipids in pollen, while pollen-feeder-zoophilus plants have the highest [12]. Up to 60% of fatty acids are unsaturated acids (oleic, linoleic, and linolenic), while palmitic acid is the most common among the saturated [13]. It is observed that pollen can have compounds that are toxic to some insects or even to bees, and the contents are species dependent [14]. In most cases those are alkaloids, phenolics, and cyanogenic glycosides, which role is to promote pollinators constancy, defense pollen from non-pollinators, and reduce pollinator infection with antimicrobial property [15,16,17,18]. Beside all of this, pollen contains ~20% of water, but some grasses can have up to 50% [19]. Rest of the constituents, such as sterols, vitamins (β-carotene, B1; B2; B3; B5; B6; C, H; folic acid, and E), enzymes, hormones, and terpenes are also essential for bees [20,21,22].

Besides floral scents and visual stimuli, nectar and pollen chemistry and morphology are the most common flower traits that attract bees [23,24,25]. Nectar and pollen that are used as nourishment to pollinators represent a reward to pollinators and are a key element in the plant–insect interaction [26]. The highest nutritive value for bees has pollen originating from the genera *Crocus*, *Salix*, *Papaver*, *Trifolium*, *Castanea*, *Raphanus*, *Sinapis*, *Erica,* and from fruit trees [5]. Pollen is used as a food during developmental stages in the hive, reproduction, brood rearing, body size, venom production, and longevity [27]. Pollen is also directly influencing the physiological metabolism of bees and its tolerance to pathogens and pesticides [28,29]. When collected by bees, pollen is not consumed immediately, but it is stored in beehive cells, mixed with honey, nectar, and glandular secretions, undergoes lactic fermentation, and becomes ’bee bread’ [30].

In recent years pollen has been investigated by many scientists who considered it as a natural source of healthy food, energy, and functional components for human consumption [31,32,33]. Pollen was proved to have therapeutic properties, having high antioxidative, antiinflammatory, antianaemic, anticarcinogenic, antiallergenic, antiradiation, and antitoxic capacities [34,35,36]. Earlier, pollen was used to treat benign prostatic hyperplasia but now it is shown that pollen regulates the digestive and respiratory systems, enhances the cardiovascular system and blood vessel maintenance by preventing arteriosclerosis, has positive effects on bone tissue, building up immunity, and helps in wound healing and age-delaying [37,38,39]. Even Al-Salem et al. suggested that pollen has positive effect on treating neuroinflammation, and thus can be used against autism [40]. Besides, *Prunus* sp. and *Rubus* sp. pollen ethanolic extracts showed antimicrobial activity against *Erwinia carotovora* (subsp. *carotovora*) and *Xanthomonas campestris* that are economically important pests of agricultural crops [41].

Sour cherry is a very important fruit species in Serbia. The total acreage is ~17,500 ha with a production of 91,659 MT. It is ranked as third in the country (after plum and apple) and sixth in the world based on tonnage [42]. The most important cultivar is the ‘Oblačinska’ sour cherry that accounts for about 85% of total Serbian production, which fruits are mostly exported as frozen or canned to Western European countries. It is proved and accepted that the ‘Oblačinska’ sour cherry is not a cultivar but a mixture of different clones that vary in many morphological, pomological, and chemical traits [43,44,45]. Sour cherry is an autogamous and entomophilous fruit species, which means that insects are needed to transfer pollen to stigma in order to obtain sufficient quantity and quality of sour cherry fruits. Therefore, bearing in mind the complex chemical composition of pollen, the aim of this study was to analyze and compare the sugars, sugar alcohols, and phenolic content of pollen grains obtained from different ‘Oblačinska’ sour cherry clones in two years. As far as we know, this is the first study where pollen was gathered by hand from different sour cherry genotypes. Obtained data will help us create a chemical fingerprint of this important sour cherry cultivar, which can be further used as product rich in bioactive compounds and used as a functional food.

## 2. Materials and Methods

### 2.1. Sample Collection

Pollen of 15 different ‘Oblačinska’ sour cherry clones were collected from the Experimental Station ‘Radmilovac’, which is part of the Faculty of Agriculture, University of Belgrade, Serbia. The orchard was located 8 km north-east of Belgrade (44°45′ N; 20°35′ E, at 135 m altitude). Orchard planting and orchard maintenance are described in Guffa et al. [45]. Each clone in the orchard was represented by a single tree.

Pollen was gathered in two consecutive years (2015 and 2016). At the balloon stage (code 59, BBCH (in German: Biologische Bundesanstalt, Bundessortenamt und CHemische Industrie) scale [46]), which in the temperate region starts at the beginning of April, twigs with flowers from three scaffolds (with different orientation) from each ‘Oblačinska’ sour cherry clone were collected, transported to the laboratory, placed in jars with water, and kept at room temperature (22 ± 2 °C). From unopened flowers, anthers were collected in Petri dishes just before dehiscence. Unopened anthers were dried at room temperature for 24 h until shedding of pollen started. Afterwards, the closed dish was moved for 2 to 3 min left-and-right, in circles, and up-and-down, by hand, to cause vibration throughout the whole area, in order to increase anther breakage and pollen releasing. Empty anthers were removed with a dissecting needle. After, Petri dishes with just pollen inside were kept frozen at −18 °C until chemical analysis.

### 2.2. Chemicals

Acetonitrile and formic acid (both MS grade), methanol (high-performance liquid chromatography, HPLC grade), sodium hydroxide, sodium acetate, and hydrochloric acid were purchased from Merck (Darmstadt, Germany). Ultra-pure water (Thermofisher TKA MicroPure water purification system, 0.055 µS/cm; Bremen Germany) was used to prepare standard solutions and blanks. The solid phase extraction (SPE) cartridges used to concentrate the samples were Strata C18–E (500 mg/3 mL) obtained from Phenomenex (Cluster d.o.o., Belgrade, Serbia). Syringe filters (25 mm, nylon membrane, 0.45 µm) were purchased from Psi lab d.o.o. (Belgrade, Serbia). Filter paper (Whatman No.1) was supplied by Merck.

Phenolic standards (protocatechuic acid, *p*-hydroxybenzoic acid, vanillic acid, syringic acid, ellagic acid, chlorogenic acid, caffeic acid, *p*-coumaric acid, ferulic acid, sinapic acid, cinnamic acid, rutin, hyperoside, cynaroside, apiin, naringin, astragalin, catechin, luteolin, apigenin, naringenin, kaempferol, aesculin, phloridzin, coniferyl aldehyde, and aesculetin) were supplied by Sigma Aldrich (Steinheim, Germany).

Sugar standards (trehalose, arabinose, glucose, fructose, sucrose, isomaltotriose, turanose maltose, and maltotriose) were purchased from Tokyo Chemical Industry (TCI, Europe, Belgium). Standard of sorbitol was obtained from Sigma Aldrich (Steinheim, Germany).

### 2.3. Sample Preparation

Pollen from each clone (0.5 g) was measured on an analytical balance and suspended in 10 mL of methanol/water (containing 5% formic acid; 7:3, *v*/*v*). After 1 h on the ultrasonic bath, the resulting mixture was centrifuged at 4500 rpm. The solution was concentrated under vacuum at 40 °C until methanol was eliminated. To the residual aqueous extract, 0.1% solution of hydrochloric acid was added to a final volume of 10 mL. This solution was further purified through a SPE column, which was previously conditioned with 3 mL of methanol and 9 mL of ultra-pure water. After applying to the SPE column, the sample was washed with 6 mL of ultra-pure water to remove all residual sugars and other polar compounds. The aqueous fraction was used for the determination of sugars using HPAEC-PAD system. The phenolic fraction was eluted from a cartridge with a solution of 1.5 mL of acidified methanol (0.1% HCl solution). The resulting methanol solutions were stored at −20 °C until analyzed. The extracts were filtered through a 0.45 μm nylon membrane filter prior to UHPLC-DAD MS/MS analysis.

### 2.4. Analysis of Carbohydrate Content

For the quantification of sugars and sugar alcohols the HPAEC-PAD system was used. Carbohydrates were analyzed in pollen samples on a Carbo PacPA10 pellicular anion-exchange column (4 × 250 mm; Dionex, Sunnyvale, CA, USA) at 30 °C. Each sample (25 µL) was injected with an ICS AS-DV 50 autosampler (Dionex, Sunnyvale, CA, USA). Carbohydrates were eluted with a flow rate set to 0.7 mL/min, in gradient prepared from 600 mM sodium hydroxide (eluent A), 500 mM sodium acetate (eluent B), and ultrapure water (eluent C). The gradient program was as follows: 0.0–20.0 min, 15% A; 20.1–30.0 min, 20% A; 0.0–5.0 min, 0% B; 5.1–12.0 min, 2% B; 12.1–20.0 min, 4% B; and 20.1–30.0 min, 20% B. The calibration of carbohydrates was performed with standard solutions of sugars and sugar alcohols. Table 1 provides data on limit of detection (LOD), limit of quantification (LOQ), and recovery (R, %).

### 2.5. Determination of Individual Polyphenols

For the quantification of phenolic compounds the UHPLC-DAD MS/MS system was used. Elution was done on 40 °C, using mobile phase water + 0.1% acetic acid (A) and acetonitrile (B) on Syncronis C18 column, in the following concentration gradient: 5% B, 2.0 min; 5–95% B, 2.0–12.0 min; 95–5% B, 12.0–12.2 min; and 5% B to 15 min. The mobile phase flow was set to 0.3 mL/min, and wavelengths were 254 and 280 nm. Injection volume was 5 µL.

A mass spectrometer was equipped with a heated electrospray ionization source with the vaporizer temperature kept at 200 °C, with a spray voltage of 5 kV and capillary temperature of 300 °C. The mass spectrometry data were acquired in the negative ion mode, in the *m*/*z* range from 100 to 1000. Multiple mass spectrometric scanning modes, including full scanning (FS), and product ion scanning (PIS), were conducted for the qualitative analysis of the targeted compounds. The collision-induced fragmentation experiments were performed using argon as the collision gas, and the collision energy was varied depending on the compound. For the quantitative analysis of phenolic compounds the time-selected reaction monitoring (tSRM) experiments were performed for each standard compound. The molecular ions and the two most intense fragments from the MS^2^ spectrum were previously defined as dominant in the PIS experiments (Table 2). Table 2 also provides the LOD, LOQ, and correlation coefficient.

Xcalibur software (version 2.2; Thermo Fisher Scientific, Bremen, Germany) was used to control the instrument [44]. Polyphenols were quantified in pollen by comparing with commercial standards. Only the MS/MS peak areas were used for quantification and calibration curves for each standard were obtained. The total content of each compound was calculated by comparing the peak area with the peak area of the corresponding standard, and were expressed as mg/kg.

### 2.6. Statistical Analysis

Data of all measurements presented in the tables are the mean of three replicates ± standard deviation. Tukey’s test was used to detect the significance of differences (*p* ≤ 0.05) between mean values. Statistical analyses were performed using the NCSS program (www.ncss.com) [44]. Principal component analysis was performed using the PLS_ToolBox software package for MATLAB (Version 7.12.0; (Eigenvector Research, Inc., Wenatchee, WA, USA) [44].

## 3. Results and Discussion

### 3.1. Carbohydrate Profile

The content of carbohydrates in 15 pollen samples is shown in Table 3 as a mean value for 2015 and 2016. Glucose was the most abundant sugar, followed by fructose, sucrose, and sugar alcohol sorbitol. According to the obtained average values of all analyzed saccharides, the sum of the glucose, fructose, and sucrose in investigated pollen samples was from 86.18 to 93.46% (cones VIII/1 and II/10, respectively). Based on the total content of the analyzed sugars and sugar alcohols, primarily genotypes I/1 (324.86 mg/g), and XIII/1 (283.65 mg/g) had the largest amounts. The glucose/fructose ratios were also calculated, as presented in Table 3. The two monosaccharide concentrations had roughly equal amounts in clones III/9, IV/1, V/P, and X/2, which was expected since sucrose synthase and invertase digests sucrose to glucose and fructose [47]. Other pollen samples were characterized with ratios above 1.0, even one pollen sample (VII/2P) had the G/F ratio 1.8 (Table 3). The content of dominant sugar components, glucose, fructose, and sucrose varied significantly among investigated clones, indicating high variability. No matter that it is already proven that fructose and glucose concentrations in pollen vary according to the botanical origin [48], in this study their level was genetically dependent.

Glucose was the most abundant sugar and it was the largest in clone I/1 (136.71 mg/g). Clone IX/1 stored a high concentration of glucose (132.49 mg/g). The contents of fructose and sucrose varied in the range from 56.72 (VII/2P) to 92.73 mg/g (I/1), and from 31.92 (IX/1) to 75.11 mg/g (II/2), respectively. Besides being important for pollen germination in reproductive process, sucrose in pollen is crucial for bee-learning processes and memory formation in foraging choice [49].

For minor sugar components, such as trehalose, arabinose, isomaltose, turanose, maltose, and maltotriose, some variations were found among clones. For trehalose a range of concentrations was measured from 0.93 (in II/2) to 5.31 mg/g (in VIII/1). Trehalose is a product of the activity of trehalose-6-phopahet synthase as a response to hydration and desiccation. The fact that up to five times more trehalose was detected in several genotypes potentially can be associated with prolonged drought of those genotypes [50]. Interestingly, pollen sample VIII/1 was reported to have the highest content of maltotriose (0.54 mg/g), but the lowest concentrations of arabinose (0.17 mg/g) and isomaltose (0.48 mg/g). Further, sorbitol was found in the range from 7.12 (VII/2P) to 22.38 mg/g (VIII/1), and it was almost as large as in the sample IX/P.

### 3.2. Phenolic Profile

Phenolic compounds, which are very variable in pollen grains, are the most important bioactive substances in pollen because they provide antioxidant activity, antimicrobial capability, and are responsible for the color and bitter taste of the grain [27,51]. According to Negri et al., the main constituents of pollen phenolics are flavonoid glycosides [52]. In this study, twenty-six different phenolic compounds were quantified using the available commercial standards and the results are presented in Table 4. Due to the easier explanation, phenolics were divided into five structurally different groups: (1) Benzoic acid derivatives (five compounds), (2) cinnamic acid derivatives (six compounds), (3) flavonoid glycoside (six compounds), (4) flavonoid aglycones (five compounds), and (5) other phenolics (five compounds). The sour cherry clone with the highest content of determined phenolics was IV/8 (445.57 mg/kg), while clone V/P had the lowest content of phenolic compounds (89.98 mg/kg).

*p*-Hydroxybenzoic, vanillic, and syringic acids were found in all fifteen investigated pollen samples of the sour cherry clones. Vanillic acid was found to be the dominant with concentration up to 10 mg/kg in three clones (I/1, VIII/1, and XIII/1). Bonvehĺ el al. also found vanillic acid as a very important constituent of pollen grains that is responsible for antioxidant activity [53]. Protocatechuic acid and ellagic acid were quantified in several samples mainly at low concentrations. However, the concentration of ellagic acid in clone VIII/1 (1.75 mg/kg) was significantly higher when compared with the other clones.

The results of the LC/MS analysis have shown that cinnamic acid derivatives were abundant in sour cherry pollen extracts. All derivatives, except sinapic acid and cinnamic acid, were found in considerable quantity. According to Almaraz-Abarca et al. the most common phenolic acids in pollen are chlorogenic, ferulic, cinnamic, and caffeic acids [4]. Chlorogenic acid was found to be dominant with a concentration up to 50 mg/kg in four clones (III/9, VIII/1, XIII/1, and XIV/3). The compounds, caffeic acid and *p*-coumaric acid, were found to be the most abundant in clones IV/8 (15.95 mg/kg and 51.20 mg/kg, respectively) and VIII/1 (15.35 mg/kg and 53.99 mg/kg, respectively). Some studies reveled that chlorogenic acid has positive effects on Alzheimer’s disease, obesity, and blood pressure [54]. Caffeic acid has a strong antioxidant capacity by eliminating oxygen free radicals, which in combination with chlorogenic acid has an even more powerful effect. Besides it protects α-tocopherol in low-density lipoprotein and it is a promising photoprotective agent [55]. *p*-Coumaric acid was also proved to have high antioxidative properties and protect humans from various kinds of cancer and cardiovascular diseases [56]. Ferulic acid and sinapic acid were quantified in the highest concentrations in clone IV/8 (56.70 mg/kg and 5.21 mg/kg, respectively). 

Rutin (quercetin 3-*O*-rutinoside) was the predominant compound from the group of flavonoid glycosides and the most abundant phenolic compound found in sour cherry pollen in this study. The lowest amount of rutin was found in clone V/P (50.28 mg/kg) and the highest amount was found in clone III/9 (181.12 mg/kg). This indicates high biological and nutritional quality of studied pollen due to its high antioxidant activity [57]. Generally, rutin was demonstrated to have a neuroprotective effect, sedative, anticonvulsant, analgesic, and antiarthritic activities, antidiabetic and antiosteoporotic effect, and to improve the cardiovascular, respiratory, reproductive, and gastrointestinal system [58].

Among the other investigated flavonoid glycosides, hyperoside—quercetin 3-*O*-galactoside (18.10 mg/kg and clone III/9) and astragalin—kaempferol 3-*O*-glucoside (31.17 mg/kg and clones IV/8) were found at significant concentrations compared to the other compounds in this group.

From the group of flavonoid aglycones, catechin was found in slightly higher concentration, ranging from 3.11 (clone XIV/5) to 8.87 mg/kg (clone III/9), but the most abundant was kaempferol with a concentration of 27.01 mg/kg in clone IV/8. Generally one of the main flavonols in bee pollen is kaempferol [59]. Other flavonoid aglycones were quantified in very low amounts such as luteolin, apigenin, and naringenin. The results obtained in the work of Al-Samarrai et al. indicated that date palm pollen has some level of naringin (64.574 mg/kg), and apigenin (109.117 mg/kg) too [60].

The other phenolic compound, coumarin aesculin was found in large concentrations in several clones, with the highest value of 29.25 mg/kg in clone VIII/1. In the same clone, the highest concentration of aesculetin, also coumarin derivate, was found at a concentration of 0.98 mg/kg. According to Tattini et al. the role of aesculin and aesculetin is in photoprotection, and these phenolic compounds can have the same role in pollen grains [61].

### 3.3. Principal Component Analysis

**Figure 1:**
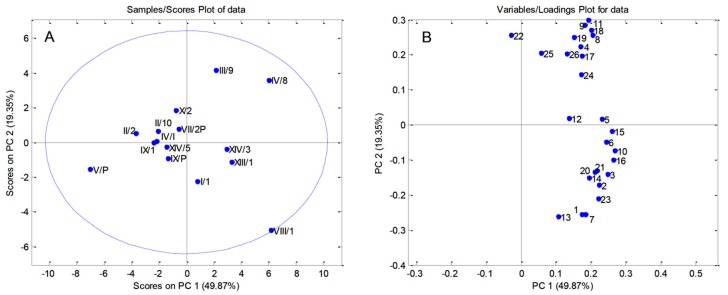
Principal component (PC) scores and loadings plot on quantified polyphenols (**A**,**B**) from pollen samples of 15 ‘Oblačinska’ sour cherry clones. Numbers on (**B**) correspond to the quantified polyphenols as given in Table 4.

**Figure 2:**
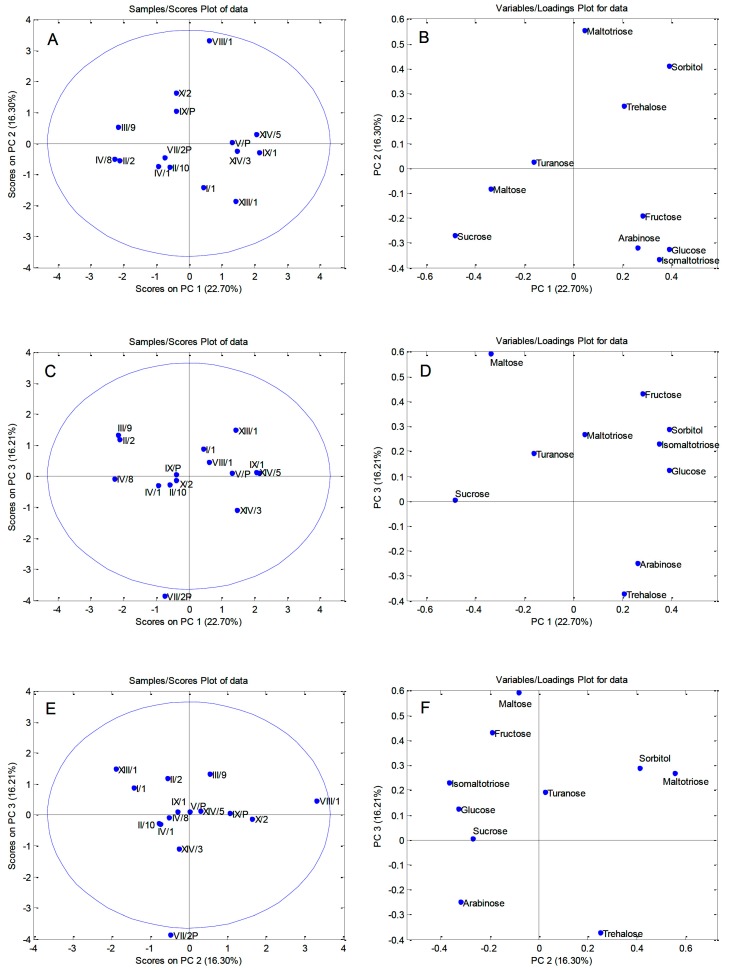
Principal component analysis (PCA) performed on sugar contents in pollen samples of ‘Oblačinska’ sour cherry clones: Scores plots of the first three principal components (**A**,**C**,**E**) and loadings plots (**B**,**D**,**F**).

A principal component analysis (PCA) was used to establish differences among pollen samples of sour cherry clones according to their chemical compositions. Two procedures were performed separately on the quantified polyphenols (Figure 1A,B), and carbohydrates (Figure 2A–F). The initial matrices 15 (the number of pollen samples from the clones) × 26 (quantified polyphenols), and 15 (the number of pollen samples) × 10 (quantified carbohydrates) were processed using the covariance matrix with autoscaling.

PCA carried out on polyphenols resulted in six PCs explaining 94.41% of the total variability. The first principal component accounted for 49.87%, the second 19.35%, and the third component 9.19% of the total variance. Although the majority of pollen samples were located in the central part of the PCA correlation plots (Figure 1A), some clones were distinguished from the other pollen samples. Clone VIII/1 separated from the other pollen samples based on its high content of many compounds (ellagic acid, cinnamic acid, aesculetin, coniferyl aldehyde, naringin, p-coumaric acid, aesculin, apiin, p-hydroxybenzoic acid, caffeic acid, protocatechuic acid, chlorogenic acid, and vanillic acid; Figure 1B). Higher contents of syringic acid, sinapic acid, and astragalin were the most important factors in distinguishing clone IV/8 from the other pollen samples, while catechin, rutin, hyperoside, and phlorizin were responsible for the separation clone III/9. On the other hand, clone V/P was separated according to lower contents of almost all quantified polyphenols.

PCA applied on sugar contents produced a five PCs model that explained 78.41% of the variation of the data set. The first principal component accounted for 22.70%, the second 16.30%, the third 16.21%, the forth 12.61%, and the fifth 10.59% of the total variance. The PCA correlations plots and loadings plots for the first three principal components are shown in Figure 2. As it can be seen from the PC1/PC2 scores plot (Figure 2A), a differentiation of the pollen samples was not possible based on the sugar contents. Pollen VIII/1 distinguished from the other samples along the PC2 axis (Figure 2A) by its higher contents of maltotriose, sorbitol, and trehalose (Figure 2B). PC1/PC3 and PC2/PC3 scores plots showed separation of clone VII/2P along the PC3 axis (Figure 2C,E). Notably lower levels of fructose and maltose are the most important factors responsible for the separation of clone VII/2P from the other samples (Figure 2D,F).

## 4. Conclusions

High sugar and phenolic compounds content (especially rutin, chlorogenic, caffeic, and *p*-coumaric acid) proved that pollen formed clones IV/8, XV/3, and VIII/1 that had high antioxidative potential. Samples of pollen analyzed in this experiment were well distinguished with a PCA analysis Based on this it was clarified that all examined clones of ‘Oblačinska’ sour cherry showed a different chemical profile. This tells us that ‘Oblačinska’ sour cherry was not a cultivar but a population of different genotypes, which showed big variability due to both generative and vegetative propagation during the last decades.

Although comparison of our results with the published literature was practically impossible, since no work on the sour cherry handpicked pollen was published so far, especially not in the way it was done in the framework of this study, but we found this investigation very important. Knowledge about its active components could have a positive influence on human health because pollen can be used as an apitherapeutic product, to support pharmacological treatment, in prevention and/or curing of diseases, or as a ‘super food’. Chemical, nutritional, and microbiological traits of ‘Oblačinska’ sour cherry pollen can give us some new beneficial usages in medicinal regimes.

## Figures and Tables

**Table 1:** Validation parameters: Retention time (*t*_R_), limit of detection (LOD), limit of quantification (LOQ), and recovery (*R*, %).

Name	*t*_R_ (min)	LOD × 10^−3^ (µg/mL)	LOQ × 10^−3^ (µg/mL)	*R* (%)
Sorbitol	2.76	0.19	0.57	102
Trehalose	3.55	0.25	0.76	109
Arabinose	4.85	0.19	0.58	98
Glucose	5.55	0.06	0.17	103
Fructose	6.41	0.08	0.24	104
Isomaltose	8.70	0.12	0.35	97
Sucrose	9.20	0.09	0.26	99
Turanose	14.90	0.05	0.16	105
Maltose	17.89	0.10	0.30	101
Maltotriose	23.12	0.15	0.48	95

**Table 2:** List of quantified phenolics in in negative ion mode: Parent ion (*m*/*z*), product ions (*m*/*z*) with specified collision energy (CE, eV), mean expected retention time (*t*_R_, min), limit of detection (LOD, mg/L) and quantification (LOQ, mg/L), and correlation coefficient (*R*^2^).

No	Compound	Parent Ion, *m*/*z*	Product Ion, *m*/*z* (CE, eV)	*t*_R_, min	LOD	LOQ	*R* ^2^
**Benzoic Acid Derivatives**							
**1**	Protocatechuic acid	153.013	108.09 (23); 109.10 (14)	4.44	0.10	0.34	0.9980
**2**	*p*-Hydroxybenzoic acid	137.057	93.19 (19); 108.33 (22)	4.68	0.14	0.48	0.9934
**3**	Vanillic acid	167.034	153.00 (15); 108.00 (21)	5.67	0.02	0.08	0.9957
**4**	Syringic acid	197.046	153.02 (16); 182.02 (21)	6.07	0.04	0.13	0.9968
**5**	Ellagic acid	300.98	284.00 (32); 300.04 (30)	6.76	0.11	0.37	0.9938
**Cinnamic Acid Derivatives**							
**6**	Chlorogenic acid	353.103	191.28 (25)	5.33	0.08	0.27	0.9980
**7**	Caffeic acid	179.004	134.00 (13); 135.00 (16)	5.82	0.11	0.38	0.9951
**8**	*p*-Coumaric acid	163.031	93.12 (39); 119.09 (16)	6.67	0.12	0.41	0.9947
**9**	Ferulic acid	193.057	134.00 (18); 178.00 (15)	6.93	0.15	0.50	0.9933
**10**	Sinapic acid	223.082	149.21 (36)	6.95	0.08	0.26	0.9984
**11**	Cinnamic acid	147.050	103.00 (10); 129.00 (10)	8.73	0.07	0.22	0.9991
**Flavonoid Glycosides**							
**12**	Rutin	609.197	299.98 (42); 301.20 (32)	6.42	0.09	0.31	0.9976
**13**	Hyperoside	463.100	300.00 (30); 271.00 (43)	6.67	0.10	0.34	0.9976
**14**	Cynaroside	447.090	285.00 (30)	6.91	0.12	0.40	0.9975
**15**	Apiin	563.140	465.00 (25); 269.00 (46)	6.97	0.11	0.38	0.9956
**16**	Naringin	579.241	151.42 (43); 217.26 (33)	7.01	0.16	0.53	0.9937
**17**	Astragalin	447.090	284.00 (33)	7.03	0.14	0.46	0.9975
**Flavonoid Aglycones**							
**18**	Catechin	289.094	203.00 (23); 245.03 (31)	5.41	0.14	0.45	0.9953
**19**	Luteolin	285.035	133.05 (30); 150.95 (24)	8.45	0.10	0.32	0.9958
**20**	Apigenin	269.032	117.24 (43); 149.00 (24)	9.12	0.11	0.36	0.9981
**21**	Naringenin	271.036	119.10 (25); 151.07 (19)	9.22	0.09	0.28	09974
**22**	Kaempferol	285.074	211.00 (32); 227.00 (32)	9.39	0.04	0.13	0.9961
**Other Phenolics**							
**23**	Aesculin	339.080	133.09 (44); 177.06 (25)	4.95	0.01	0.05	0.9999
**24**	Phlorizin	435.149	273.16 (20); 167.16 (34)	7.37	0.05	0.15	0.9978
**25**	Coniferyl aldehyde	177.060	162.00 (17); 97.00 (14)	7.70	0.03	0.08	0.9968
**26**	Aesculetin	176.992	133.28 (19); 105.25 (20)	7.71	0.10	0.34	0.9969

**Table 3:** The average (2015 and 2016) contents of carbohydrates (mg/g), in pollen of 15 ‘Oblačinska’ sour cherry clones.

Carbohydrates/Clones	I/1	II/2	II/10	III/9	IV/1	IV/8	V/P	VII/2P	VIII/1	IX/1	IX/P	X/2	XIII/1	XIV/3	XIV/5
**Sorbitol**	16.00 ^e^	14.03 ^d^	9.87 ^b^	11.28 ^c^	7.22 ^a^	8.01 ^a^	17.41 ^f^	7.12 ^a^	22.38 ^h^	16.68 ^e,f^	21.03 ^g^	14.96 ^d^	14.83 ^d^	17.30 ^f^	17.24 ^f^
**Trehalose**	4.03 ^e^	0.93 ^a^	1.58 ^b^	2.36 ^c^	3.54d ^e^	3.19 ^c,d^	2.98 ^c^	4.73 ^f^	5.31 ^g^	2.50 ^c^	1.84 ^b^	2.36 ^c^	1.52 ^b^	4.23 ^e,f^	4.56 ^f^
**Arabinose**	0.35 ^b^	0.48 ^c^	0.30 ^b^	0.23 ^a,b^	0.46 ^c^	0.20 ^a^	0.64 ^d^	0.49 ^c^	0.17 ^a^	0.34 ^b^	0.28 ^b^	0.18 ^a^	0.38 ^b^	0.74 ^e^	0.49 ^c^
**Glucose (G)**	136.71 ^k^	89.77 ^b^	116.71 ^h^	86.44 ^a^	95.69 ^d^	91.46 ^c^	95.44 ^d^	102.38 ^e^	107.47 ^f^	132.49 ^j^	92.61 ^c^	85.83 ^a^	129.51 ^i^	95.42 ^d^	110.68 ^g^
**Fructose (F)**	92.73 ^j^	68.57 ^b^	83.52 ^f^,^g^	85.04 ^h^	87.45 ^i^	71.60 ^c^	91.69 ^j^	56.72 ^a^	74.70 ^d^	82.17 ^e,f^	67.25 ^b^	81.31 ^e^	86.96 ^i^	82.46 ^e,f^	84.45 ^g,h^
**Sucrose**	66.40 ^j^	75.11 ^k^	56.12 ^g^	73.97 ^k^	50.75 ^f^	59.38 ^h^	38.58 ^b^	63.84 ^i^	38.64 ^b^	31.92 ^a^	36.68 ^b^	42.58 ^c^	41.83 ^c^	46.88 ^e^	44.78 ^d^
**Isomaltotriose**	0.91 ^e^	1.16 ^f^	0.49 ^a^	0.78 ^d^	0.67 ^c^	0.94 ^e^	0.92 ^e^	0.69 ^c,d^	0.48 ^a^	1.34 ^g^	0.65 ^c^	0.56 ^b^	1.92 ^i^	1.20 ^f^	1.81 ^h^
**Turanose**	0.38 ^c^	0.39 ^c,d^	0.16 ^a^	0.44 ^d^	0.60 ^f^	0.85 ^g^	0.27 ^b^	0.28 ^b^	0.47 ^d,e^	0.35 ^b,c^	0.60 ^f^	0.36 ^b,c^	0.54 ^e,f^	0.38 ^c^	0.55 ^e,f^
**Maltose**	7.17 ^e^	8.60 ^f^	5.27 ^c^	7.48 ^e^	6.27 ^d^	6.54 ^d^	5.64 ^c^	2.31 ^a^	6.05 ^c^	3.84 ^b^	5.61 ^c^	4.23 ^b^	5.94 ^c^	4.36 ^b^	4.02 ^b^
**Maltotriose**	0.18 ^a^	0.38 ^c^	0.26 ^b^	0.47 ^d^	0.24 ^b^	0.18 ^a^	0.32 ^c^	0.19 ^a^	0.54 ^e^	0.30 ^b,c^	0.20 ^a^	0.40 ^c,d^	0.22 ^a,b^	0.28 ^b,c^	0.44 ^d^
**G/F ratio**	1.47	1.31	1.40	1.02	1.09	1.28	1.04	1.81	1.44	1.61	1.38	1.06	1.49	1.16	1.31
**Total**	324.86	259.42	274.28	268.49	252.89	242.35	253.89	238.75	256.21	271.93	226.75	232.77	283.65	253.25	269.02

Different letter in the same row denotes a significant difference between clones according to the Tukey’s test, *p* < 0.05.

**Table 4:** The average (2015 and 2016) contents of phenolic compounds (mg/kg), in pollen of 15 ‘Oblačinska’ sour cherry clones.

No	Phenolic Compounds/Clones		I/1	II/2		II/10	III/9	IV/1	IV/8	V/P	VII/2P	VIII/1	IX/1	IX/P	X/2	XIII/1	XIV/3	XIV/5
		**Benzoic Acid Derivatives**																
1	Protocatechuic acid		0.42 ^d^	–		–	–	–	0.10 ^b^	–	0.11 ^b^	0.54 ^e^	0.04 ^a^	0.12 ^b^	0.08 ^a,b^	0.34 ^c^	0.64 ^f^	0.04 ^a^
2	*p*-Hydroxybenzoic acid		4.45 ^e^	1.70 ^b^		2.91 ^c^	3.62 ^d^	2.37 ^c^	5.57 ^f^	0.75 ^a^	3.63 ^d^	7.80 ^g^	2.46 ^c^	3.62 ^d^	2.86 ^c^	7.85 ^g^	5.50 ^f^	3.81 ^d,e^
3	Vanillic acid		10.68 ^g^	1.52 ^b^		1.75 ^b^	2.16 ^c^	2.58 ^c^	3.47 ^d^	0.57 ^a^	4.54 ^e^	10.68 ^g^	1.35 ^b^	2.16 ^c^	1.39 ^b^	10.92 ^g^	7.74 ^f^	2.82 ^c^
4	Syringic acid		0.65 ^b^	0.58 ^b^		0.61 ^b^	0.95 ^e^	0.64 ^b^	1.22 ^f^	0.20 ^a^	0.55 ^b^	0.65 ^b^	0.54 ^b^	0.73 ^c^	0.80 ^d^	0.72 ^c^	0.72 ^c^	0.59 ^b^
5	Ellagic acid		–	0.37 ^d^		–	–	–	–	0.02 ^a^	0.04 ^a^	1.75 ^e^	0.17 ^b^	0.08 ^a^	0.28 ^c^	–	0.06 ^a^	–
	Total		16.20	4.17		5.27	6.73	5.59	10.36	1.54	8.87	21.42	4.56	6.71	5.41	19.83	14.66	7.26
		**Cinnamic Acid Derivatives**																
6	Chlorogenic acid		43.70 ^f^	29.28 ^c^		23.64 ^b^	59.93 ^j^	34.24 ^d^	42.57 ^f^	7.27 ^a^	37.23 ^e^	58.93 ^j^	33.21 ^d^	38.46 ^e^	47.66 ^g^	53.33 ^h^	56.85 ^i^	24.92 ^b^
7	Caffeic acid		5.51 ^c^	1.99 ^a^		3.56 ^b^	5.03 ^c^	3.18 ^b^	15.95 ^f^	1.36 ^a^	3.78 ^b^	15.35 ^f^	2.99 ^b^	3.98b ^c^	4.23 ^c^	6.17 ^d^	12.94 ^e^	3.46 ^b^
8	*p*-Coumaric acid		29.89 ^e^	10.52 ^b^		19.84 ^c^	26.54 ^d^	18.19 ^c^	51.20 ^h^	4.82 ^a^	24.58 ^d^	53.99 ^h^	18.60 ^c^	23.49 ^d^	18.45 ^c^	42.94 ^g^	39.03 ^f^	24.29 ^d^
9	Ferulic acid		35.22 ^g^	10.68 ^b^		19.01 ^d^	24.83 ^f^	16.81 ^c^	56.70 ^i^	7.11 ^a^	21.99 ^e^	43.93 ^h^	16.87 ^c^	18.71 ^d^	21.68 ^e^	43.60 ^h^	42.46 ^h^	20.76 ^d,e^
10	Sinapic acid		1.79 ^d,e^	–		–	1.59 ^c,d^	–	5.21 ^f^	0.22 ^a^	–	–	–	–	0.83 ^b^	1.42 ^c^	2.28 ^e^	–
11	Cinnamic acid		2.44 ^g^	0.59 ^b^		0.90 ^c^	1.04 ^c^	1.22 ^d^	1.61 ^e^	0.19 ^a^	1.18 ^d^	2.75 ^h^	0.99 ^c^	1.00 ^c^	1.00 ^c^	1.95 ^f^	1.54 ^e^	1.16 ^d^
	Total		118.55	53.06		66.95	118.96	73.64	173.24	20.97	88.76	174.95	72.66	81.66	93.85	149.41	155.10	74.59
		**Flavonoid Glycosides**																
12	Rutin		92.96 ^b^	93.51 ^b^		118.96 ^e^	181.12 ^i^	113.01 ^d^	157.95 ^h^	50.28 ^a^	114.38 ^d^	112.89 ^d^	106.34 ^c^	117.42 ^e^	139.47 ^f^	119.42 ^e^	144.43 ^g^	107.03 ^c^
13	Hyperoside		9.68 ^b^	9.44 ^b^		11.07 ^c^	18.10 ^d^	11.08 ^c^	17.85 ^b^	4.82 ^a^	12.07 ^c^	11.05 ^c^	10.44 ^c^	12.15 ^c,d^	13.31 ^d^	11.66 ^c^	13.91 ^d^	11.03 ^c^
14	Cynaroside		6.14 ^f^	0.34 ^a^		–	1.15 ^c^	0.76 ^a,b^	5.22 ^e^	–	0.91 ^b^	–	2.41 ^d^	–	–	5.83 ^e,f^	2.58 ^d^	0.91 ^b^
15	Apiin		0.08 ^c^	0.01 ^a^		0.04 ^b^	0.04 ^b^	–	0.09 ^c^	–	0.05 ^b^	0.16 ^d^	0.04 ^b^	0.05 ^b^	0.03 ^a,b^	–	0.06 ^b^	0.05 ^b^
16	Naringin		0.22 ^c^	0.08 ^a^		0.14 ^b^	0.17 ^b^	0.13 ^b^	0.28 ^d^	0.05 ^a^	0.15 ^b^	0.34 ^e^	0.14 ^b^	0.16 ^b^	0.17 ^b^	0.24 ^c^	0.21 ^c^	0.15 ^b^
17	Astragalin		15.04 ^b^	14.05 ^b^		18.32 ^d^	22.82 ^e^	14.17 ^b^	31.17 ^f^	7.40 ^a^	15.38 ^b^	16.90 ^b^	14.75 ^b^	16.98 ^b^	20.42 ^d,e^	16.86 ^b^	19.10 ^d^	14.84 ^b^
	Total		124.12	117.43		148.53	223.40	139.15	212.56	62.55	142.94	141.34	134.12	146.76	173.40	154.01	180.29	134.01
		**Flavonoid Aglycones**																
18	Catechin		5.07 ^bc^	4.98 ^bc^		4.47 ^b^	8.87 ^d^	4.63 ^b^	5.99 ^c^	–	5.62 ^c^	5.07 ^bc^	4.57 ^b^	4.90 ^bc^	5.58 ^c^	5.20 ^bc^	5.21 ^bc^	3.11 ^a^
19	Luteolin		0.26 ^b^	0.35 ^d^		0.40 ^e^	0.44 ^e^	–	0.31 ^c^	0.20 ^a^	0.34 ^d^	–	0.30 ^c^	–	0.43 ^e^	0.40 ^e^	–	0.32 ^c^
20	Apigenin		0.08 ^a^	0.10 ^b^		0.11 ^b^	0.17 ^d^	0.11 ^b^	0.17 ^d^	0.07 ^a^	0.18 ^d^	0.16 ^cd^	0.12 ^b^	0.07 ^a^	0.11 ^b^	0.15 ^c^	0.15 ^c^	0.17 ^d^
21	Naringenin		0.07 ^b^	0.13 ^d^		0.12 ^d^	0.18 ^ef^	0.10 ^c^	0.18 ^ef^	0.16 ^de^	0.21 ^f^	0.13 ^d^	0.12 ^d^	0.04 ^a^	0.13 ^d^	0.13 ^d^	0.15 ^de^	0.10 ^c^
22	Kaempferol		0.97 ^b^	0.95 ^b^		1.53 ^g^	1.59 ^g^	1.16 ^bc^	27.01 ^h^	0.68 ^a^	1.09 ^bc^	1.24 ^d^	0.97 ^b^	1.18 ^c^	1.32 ^e^	1.18 ^c^	1.31 ^e^	1.38 ^f^
	Total		6.45	6.51		6.63	11.25	6.00	33.66	1.11	7.44	6.60	6.08	6.19	7.57	7.06	6.82	4.76
		**Other Phenolics**																
23	Aesculin		9.53 ^c^	7.61 ^b^		9.74 ^c^	12.61 ^d^	9.93 ^c^	13.45 ^d^	3.43 ^a^	11.31 ^d^	29.25 ^f^	9.31 ^c^	10.52 ^c^	10.17 ^c^	12.50 ^d^	17.14 ^e^	9.38 ^c^
24	Phlorizin		0.51 ^b^	0.78 ^c^		0.75 ^c^	1.63 ^f^	1.00 ^de^	0.99 ^de^	0.25 ^a^	0.94 ^d^	0.89 ^d^	0.51 ^b^	0.59 ^b^	1.04 ^e^	0.93 ^d^	1.08 ^e^	0.79 ^c^
25	Coniferyl aldehyde		0.42 ^d^	0.16 ^b^		0.39 ^cd^	0.56 ^e^	0.31 ^c^	0.75 ^f^	0.08 ^a^	0.39 ^cd^	1.19 ^g^	0.27 ^bc^	0.51 ^e^	0.23 ^b^	1.13 ^g^	0.26 ^bc^	0.53 ^e^
26	Aesculetin		0.32 ^d^	0.14 ^b^		0.30 ^d^	0.47 ^e^	0.25 ^cd^	0.59 ^f^	0.07 ^a^	0.32 ^d^	0.98 ^g^	0.24 ^cd^	0.44 ^e^	0.16 ^b^	0.94 ^g^	0.20 ^bc^	0.43 ^e^
	Total		10.78	8.69		11.18	15.27	11.49	15.78	3.80	12.96	32.31	10.33	12.06	11.60	15.50	18.68	11.13
	Total of all		276.09	189.84		238.55	375.62	235.87	445.57	89.98	260.96	376.62	227.73	257.34	291.83	345.78	375.53	232.07

Different letter in the same row denotes a significant difference between clones according to the Tukey’s test, *p* < 0.05; “–”stands for not detected compound.
